# Impact of Vitamin D Deficiency on Ischemic Stroke Severity: Insights from a Prospective Study

**DOI:** 10.7759/cureus.69376

**Published:** 2024-09-13

**Authors:** Jibin Simon, Tirumalasetty Sriharsha, Ananthakumar Perumal Kumaresan, Utham Chand, Sharan Bose

**Affiliations:** 1 Internal Medicine, Saveetha Medical College and Hospital, Saveetha Institute of Medical and Technical Sciences, Saveetha University, Chennai, IND

**Keywords:** acute ischemic stroke (ais), correlation of vitamin d and severity of stroke, initial stroke severity scoring, stroke outcome, vitamin d deficiency

## Abstract

Background

Ischemic stroke, characterized by the obstruction of blood flow to the brain, is a major cause of morbidity and mortality worldwide. The severity of ischemic stroke is commonly assessed using the National Institutes of Health Stroke Scale (NIHSS), which helps predict patient outcomes. Recent research suggests a potential link between low vitamin D levels and an increased risk of cerebrovascular events, including ischemic stroke. However, the specific relationship between vitamin D deficiency and stroke severity remains underexplored.

Objectives

The study aimed to investigate the correlation between serum vitamin D levels and NIHSS scores in patients with ischemic stroke to determine whether vitamin D deficiency is associated with the severity of neurological deficits in these patients.

Materials and methods

This prospective observational study was conducted at Saveetha Medical College, Chennai, and involved 86 patients presenting with acute ischemic stroke. Inclusion criteria were age ≥18 years, a confirmed diagnosis of acute ischemic stroke by neuroimaging, and presentation within 24 hours of symptom onset. Exclusion criteria included hemorrhagic stroke, conditions affecting vitamin D metabolism, and current vitamin D supplementation. Serum 25-hydroxyvitamin D (25(OH)D) levels were measured using chemiluminescence immunoassay (CLIA), and NIHSS scores were assessed within 24 hours of admission. Statistical analyses included Pearson’s correlation and multivariate linear regression to adjust for confounding variables.

Results

The study found that lower serum 25(OH)D levels were correlated with higher severity of stroke symptoms, as indicated by a significant negative correlation between 25(OH)D levels and NIHSS scores at admission (Pearson correlation coefficient r = -0.4081, p < 0.001). Multivariate regression analysis confirmed this association (β = -0.3994, p < 0.001) after adjusting for age, sex, and comorbidities, with p < 0.05 considered statistically significant. In addition, age (β = 0.1123, p = 0.009) and comorbid conditions (β = 0.9565, p = 0.008) were significantly associated with higher NIHSS scores.

Conclusion

The study demonstrates a significant negative correlation between serum 25-hydroxyvitamin D levels and ischemic stroke severity, suggesting that higher vitamin D levels may be associated with less severe strokes. Further research is needed to explore the mechanistic pathways and therapeutic potential of vitamin D in stroke management. Emphasizing the importance of maintaining adequate vitamin D levels could be crucial for potentially reducing stroke severity and improving patient outcomes.

## Introduction

Ischemic stroke, characterized by the obstruction of cerebral blood flow, remains a leading cause of morbidity and mortality globally. The prognosis of ischemic stroke is influenced by multiple factors, including etiology, the location and extent of the infarct, patient age, sex, obesity, and other comorbidities [1]. Among the available tools for assessing stroke severity and predicting outcomes, the National Institutes of Health Stroke Scale (NIHSS) is the gold standard. This scale provides a systematic approach to evaluating the degree of neurological impairment, thereby assisting clinicians in making informed decisions regarding patient management and prognostication [2].

In recent years, there has been growing interest in the role of vitamin D (calciferol) in various health outcomes beyond its established function in calcium homeostasis and bone metabolism. Vitamin D, a fat-soluble vitamin that functions similarly to a steroid hormone, is synthesized in the skin upon exposure to ultraviolet B (UVB) radiation. Once produced or ingested, vitamin D binds to the vitamin D receptor (VDR), initiating a cascade of biological processes critical for maintaining health [3]. Traditionally associated with bone health, vitamin D is now being explored for its potential neuroprotective effects, particularly in skeletal muscle function and central nervous system health [4]. In addition, vitamin D influences various physiological processes, including immune modulation and cardiovascular regulation [5].

Emerging research suggests a potential link between low vitamin D levels and an increased risk of cerebrovascular events, including ischemic stroke. Several studies have indicated that vitamin D deficiency may exacerbate stroke risk factors, such as hypertension and atherosclerosis, thereby increasing the likelihood of cerebrovascular incidents [6]. Despite these findings, the specific relationship between vitamin D deficiency and ischemic stroke severity, as measured by the NIHSS, remains underexplored. This gap in the literature underscores the need for further investigation to determine whether vitamin D levels could serve as a modifiable risk factor or prognostic marker for ischemic stroke outcomes.

This prospective study investigates the correlation between vitamin D levels and NIHSS scores in patients with ischemic stroke. By examining this relationship, we aim to determine whether vitamin D deficiency is associated with the severity of neurological deficits observed in stroke patients. Elucidating this potential link could have significant implications for stroke prevention and management, emphasizing the importance of vitamin D in maintaining neurological health and potentially informing therapeutic strategies to mitigate stroke severity.

## Materials and methods

Study design and setting

This prospective observational study was conducted at Saveetha Medical College, Chennai, a tertiary care hospital, to evaluate the correlation between vitamin D levels and the severity of ischemic stroke as assessed by the NIHSS score.

Study population

Patients presenting with acute ischemic stroke to the emergency department were screened for eligibility based on predefined inclusion and exclusion criteria.

Sample size

A sample size of 86 was chosen to provide sufficient statistical power and precision, ensuring reliable and generalizable results while balancing practical feasibility.

Inclusion criteria

Eligible participants included those aged 18 years or older, with a diagnosis of acute ischemic stroke confirmed by neuroimaging modalities such as computed tomography (CT) or magnetic resonance imaging (MRI), who presented within 24 hours of symptom onset.

Exclusion criteria

Exclusion criteria included patients with a known history of conditions that affect vitamin D metabolism, such as chronic kidney disease or parathyroid disorders, as well as those with a previous history of ischemic stroke, hemorrhagic stroke, venous thrombosis, or transient ischemic attack. Patients who were taking vitamin D supplements were also excluded from the study.

Data collection

Upon admission, demographic data, medical history, and clinical characteristics were recorded. Blood samples were collected to measure serum 25(OH)D levels using chemiluminescence immunoassay (CLIA). A certified neurologist assessed the NIHSS score within 24 hours of admission to quantify stroke severity.

Vitamin D measurement

Serum 25(OH)D levels were categorized into three groups: deficient (<20 ng/mL), insufficient (20-29 ng/mL), and sufficient (≥30 ng/mL).

Statistical analysis

The collected data were analyzed using IBM SPSS Statistics for Windows, version 25.0 (IBM Corp., Armonk, NY). Descriptive statistics were employed to summarize the demographic and clinical characteristics of the study population. Continuous variables were expressed as mean ± standard deviation (SD), while categorical variables were presented as frequencies and percentages.

The correlation between serum 25(OH)D levels and NIHSS scores was evaluated using Pearson’s correlation coefficient for normally distributed variables. A p-value of <0.05 was considered statistically significant. In addition, multivariate linear regression analysis was performed to adjust for potential confounders, including age, sex, comorbid conditions, and time to presentation.

Ethical considerations

The study protocol was approved by the Institutional Ethics Committee of Saveetha Medical College (approval no. 022/03/2023/IEC/SMCH). Informed consent was obtained from all participants or their legal guardians before enrollment in the study.

## Results

Table 1 presents the age-group distribution of the 86 participants in the study, categorized into four groups: under 50, 51-60, 61-70, and over 70 years. The data indicate that the highest proportion of participants falls within the 51-60 age group (30.2%, n = 26), followed by equal distributions in the under 50 and over 70 age groups (24.4%, n = 21 each). The smallest proportion of participants is in the 61-70 age group (20.9%, n = 18). The overall age distribution demonstrates a balanced representation across the different age ranges, ensuring that the findings apply to a wide spectrum of ages.

**Table 1:** Age-group distribution Data are presented as number (n) and percentage (%). The age groups are categorized as follows: under 50 years, 51-60 years, 61-70 years, and over 70 years. The total number of participants is 86 (100.0%).

Age range (years)	Number (n) and percentage (%)
		<50	21 (24.4%)
51-60	26 (30.2%)
61-70	18 (20.9%)
>70	21 (24.4%)
Total	86 (100.0%)

Table 2 provides the gender-wise distribution of the 86 participants in the study, revealing a higher proportion of male participants (59.3%, n = 51) compared to female participants (40.7%, n = 35). This distribution indicates a notable gender disparity, with males comprising nearly 60% of the study sample. This gender imbalance should be considered when interpreting the results, as it may affect the generalizability of the findings to the broader population.

**Table 2:** Gender-wise distribution Data are presented as number (n) and percentage (%). The distribution includes female and male participants, with the total number of participants being 86 (100.0%).

Sex	Number (n) and percentage (%)
		Female	35 (40.7%)
Male	51 (59.3%)
Total	86 (100.0%)

Table 3 details the distribution of various comorbidities among the 86 participants in the study. The most common comorbidity is the combination of diabetes mellitus and hypertension, present in 30.2% (n = 26) of the participants, followed by those with no comorbidities (29.1%, n = 25). Diabetes mellitus alone is observed in 22.1% (n = 19) of the participants, while hypertension alone is found in 17.4% (n = 15). Hypothyroidism is the least common, occurring in only 1.2% (n = 1) of the participants.

**Table 3:** Comorbidity distribution Data are presented as number (n) and percentage (%). Comorbidities are categorized into diabetes mellitus, diabetes mellitus and hypertension, hypertension, hypothyroidism, and no comorbidities. The total number of participants is 86 (100.0%).

Comorbidities	Number (n) and percentage (%)
		Diabetes mellitus	19 (22.1%)
Diabetes mellitus and hypertension	26 (30.2%)
Hypertension	15 (17.4%)
Hypothyroid	1 (1.2%)
No comorbidities	25 (29.1%)
Total	86 (100.0%)

Table 4 shows a negative correlation between the NIHSS score at admission and vitamin D levels at admission, with a Pearson correlation coefficient (r) of -0.4081. The coefficient of determination (r²) is 0.1665, indicating that 16.65% of the variation in NIHSS scores can be explained by variations in vitamin D levels. This correlation is highly statistically significant, with a p-value of less than 0.001. In addition, the covariance between NIHSS scores and vitamin D levels at admission is -5.9375, further supporting the negative relationship. The test statistic for this analysis is -4.0967, based on a sample size of 86 patients, providing additional insight into the statistical methods used to determine the correlation.

**Table 4:** Pearson correlation between NIHSS scores at admission and vitamin D levels at admission Data are represented as Pearson correlation coefficient (r), coefficient of determination (r²), covariance, and sample size (n). The p-value is considered statistically significant at p<0.05, with p<0.001 indicating a highly significant result.

Parameter	Value
Pearson correlation coefficient (r)	-0.4081
Coefficient of determination (r²)	0.1665
P-value	<0.001
Covariance	-5.9375
Sample size (n)	86

**Figure 1:**
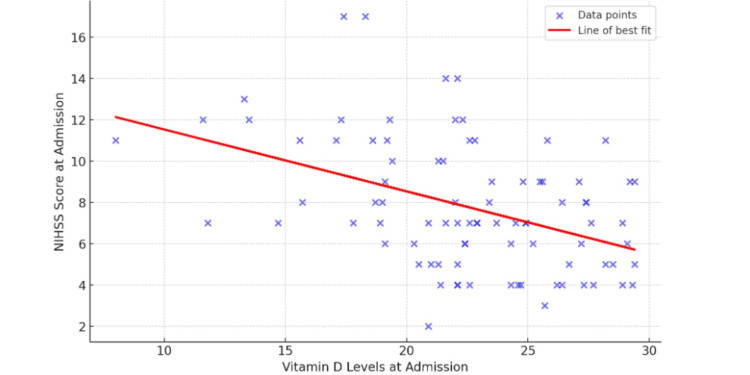
Scatter plot of NIHSS scores at admission versus vitamin D levels with line of best fit Data points: Individual patient's NIHSS scores and their corresponding vitamin D levels. Line of best fit: The regression line showing the trend between NIHSS scores and vitamin D levels. NIHSS: National Institutes of Health Stroke Scale

The scatter plot, as depicted in Figure 1, features a downward-sloping line of best fit, indicating a negative correlation between vitamin D levels and NIHSS scores at admission. These results suggest a potential impact of vitamin D levels on the severity of stroke at admission.

To further investigate the relationship between vitamin D levels at admission and NIHSS scores while adjusting for potential confounding variables, a multivariate linear regression analysis was performed, including age, sex, and comorbid conditions as covariates. In this analysis, a p-value of less than 0.05 was considered statistically significant.

The multivariate regression model demonstrated a significant negative association between vitamin D levels and NIHSS scores (β = -0.3994, p < 0.001), indicating that higher vitamin D levels are associated with lower (less severe) NIHSS scores at admission. Age was also significantly associated with NIHSS scores (β = 0.1123, p = 0.009), suggesting that older patients tend to experience more severe strokes. In addition, the presence of comorbid conditions was significantly associated with higher NIHSS scores (β = 0.9565, p = 0.008), indicating that patients with comorbidities tend to have more severe strokes. However, the association between sex and NIHSS scores was not statistically significant (β = 1.4876, p = 0.093).

## Discussion

This prospective observational study investigated the relationship between serum 25(OH)D levels and the severity of ischemic stroke as assessed by the NIHSS score. The findings indicate a significant negative correlation between vitamin D levels and NIHSS scores at admission, suggesting that higher vitamin D levels may be associated with less severe strokes. This correlation persisted even after adjusting for potential confounding variables, such as age, sex, and comorbid conditions.

Age was identified as a significant predictor of stroke severity, with older patients exhibiting more severe strokes. This relationship underscores the multifactorial nature of stroke outcomes, where age-related physiological changes and the cumulative burden of comorbid conditions play critical roles. Aging is also associated with lower endogenous production and retention of vitamin D, which may contribute to the deficiency observed in many elderly patients [7]. While age may influence vitamin D levels, our multivariate regression analysis adjusted for this factor, confirming that the independent association between low vitamin D levels and increased stroke severity remained significant. Therefore, although age is a known contributor to both vascular health and vitamin D metabolism, the observed correlation between vitamin D deficiency and stroke severity is not solely attributable to age. In addition, older adults are more likely to have multiple comorbidities, including hypertension, diabetes, and atrial fibrillation, all of which contribute to both the likelihood of experiencing a stroke and the severity of the event [8].

The correlation analysis shown in Table 4 clarifies the association between vitamin D levels and stroke severity upon admission, as determined by the NIHSS score. The modest negative correlation suggests that higher vitamin D levels may be linked to less severe stroke presentations. These findings are consistent with clinical trials that have examined the effect of supplemental vitamin D on stroke outcomes, reinforcing the established inverse relationship between stroke severity and vitamin D levels. For example, an observational case-control study by Kamal et al. (2023) concluded that vitamin D sufficiency significantly correlates with a reduced incidence of stroke, highlighting the potential role of vitamin D levels as a risk factor in acute ischemic stroke [9]. Similarly, Al Harbi et al. (2022) in a retrospective cohort study found that low vitamin D levels in patients with acute ischemic stroke were associated with greater stroke severity upon admission and reduced functional independence at discharge [10]. A meta-analysis by Gholami et al. (2019) of 25 studies involving 10,099 cases found that low serum vitamin D levels are associated with a 44% increased risk of coronary heart disease and stroke [11]. In another case-control study by Selim et al. (2019), stroke patients had significantly lower vitamin D levels than controls. Vitamin D deficiency was significantly associated with greater stroke severity, as measured by the NIHSS, and poorer outcomes at three months, assessed by the modified Rankin scale (mRS). These associations remained significant after adjusting for age, dyslipidemia, and infarction size [12].

The discussion of this study’s findings emphasizes the potential significance of vitamin D in modulating the severity of ischemic stroke. This finding aligns with broader literature identifying vitamin D as a protective factor in various cardiovascular and cerebrovascular conditions. Potential mechanisms for this protective effect include the promotion of insulin-like growth factor 1 (IGF-1) expression, which supports neuroprotection and possesses antithrombotic properties. Vitamin D may also induce vasodilation, enhance post-stroke blood flow by potentiating nitric oxide synthase (NOS), and reduce vascular stiffness, potentially lowering the risk of stroke. Its anti-inflammatory effects and ability to inhibit reactive oxygen species further protect the blood-brain barrier (BBB) after a stroke. Conversely, deficiency in 25(OH)D₃ has been linked to increased vascular stiffness, a higher risk of deep venous thrombosis, and greater stroke severity due to diminished neuroprotection and compromised cardiovascular health [13].

Upon reviewing the literature on stroke incidence and vitamin D deficiency, the Rotterdam Study provides valuable insights. It is crucial to note that although the Rotterdam Study found an association between lower vitamin D levels and stroke prevalence, it did not establish a significant link between vitamin D levels and incident stroke risk after excluding individuals with a history of stroke. Furthermore, only severe vitamin D deficiency was associated with an increased risk of stroke, emphasizing that routine vitamin D supplementation should not be considered a preventive measure for stroke without further supporting evidence [14].

Future research should explore the mechanistic pathways through which vitamin D influences stroke severity and consider longitudinal studies to assess the impact of vitamin D supplementation on stroke outcomes. Such investigations could inform clinical guidelines and therapeutic strategies aimed at optimizing vitamin D levels to potentially mitigate stroke severity and improve patient outcomes.

Limitations of the study

This study has several limitations. First, its observational design precludes the establishment of causality between vitamin D levels and stroke severity, despite adjusting for key confounders. The single-center setting may limit the generalizability of the findings to broader populations, and reliance on a single measurement of vitamin D levels at admission may not account for longitudinal variations. In addition, while the NIHSS score was used to assess stroke severity, other clinical parameters were not fully explored, potentially limiting the comprehensive understanding of vitamin D's impact on stroke outcomes.

To address these limitations, future research should consider prospective, multicenter studies with larger sample sizes to enhance generalizability. Longitudinal assessments of vitamin D levels and comprehensive outcome measures beyond NIHSS scores could provide a more nuanced understanding of vitamin D's role in stroke severity over time. In addition, randomized controlled trials investigating the effects of vitamin D supplementation on stroke outcomes would help elucidate causal relationships. Improved measurement of confounding variables, such as dietary habits and sunlight exposure, could further refine our understanding of vitamin D's impact on stroke severity.

## Conclusions

This study highlights a significant negative correlation between serum 25-hydroxyvitamin D levels and ischemic stroke severity, as measured by NIHSS scores. Despite the limitations of its observational design and single-center setting, these findings add to the growing body of evidence suggesting a potential role for vitamin D in reducing stroke severity. Future research should focus on elucidating the underlying mechanistic pathways, conducting larger-scale prospective studies, and exploring interventional strategies to further clarify vitamin D's therapeutic potential in stroke management. Such efforts may inform clinical practice and improve outcomes for stroke patients.
